# Self‐management interventions for people with multiple sclerosis: A systematic review and meta‐analysis protocol

**DOI:** 10.1002/hsr2.1536

**Published:** 2023-09-04

**Authors:** Reza Heidari‐Soureshjani, Alireza Nikbakht Nasrabadi, Masoumeh Zakerimoghadam, Tayeb Mohammadi, Arezoo Rasti

**Affiliations:** ^1^ School of Nursing and Midwifery Tehran University of Medical Sciences Tehran Iran; ^2^ Department of Biostatistics, School of Public Health Hamadan University of Medical Sciences Hamadan Iran

**Keywords:** depression and pain, fatigue, multiple sclerosis, self‐management

## Abstract

**Background and Aims:**

Educational self‐management interventions (SMI) have an important role in improving symptom management, preventing relapse of multiple sclerosis (MS) and promoting quality of life (QoL) of these patients; since there is little knowledge about overall effectiveness of MS self‐management programs and which types of SMI improves the outcomes, this research aims to assess the efficacy of structured SMI in improving health outcomes in people with MS (PwMS) by synthesizing and compare outcomes from related randomized controlled trials.

**Methods:**

In the present systematic review protocol, the keywords related to self‐management and MS will be searched in electronic databases including (PubMed, Web of Science, Scopus, EMBASE, Cochrane Central Register of Controlled Trials [CENTRAL]), gray literature resources and key journals from 2000 to July 2023. Research‐related articles will be collected and after removing duplicate articles, will be included in the study. In the screening step, titles and abstracts of articles will be reviewed and after deleting irrelevant articles, the full text of related articles will be evaluated independently by two researchers and data will be extracted from final articles and the findings will be categorized in an extraction table. Risk of bias will be assessed by using the Cochrane collaboration's tool. If possible, the data will be analyzed using random effect models and the statistical analysis will be performed using STATA software (version 14.2) developed by StataCorp.

**Discussion:**

Comparative effectiveness of SMI is currently unknown. We will analyze outcome measures used to assess effectiveness of self‐management education in improving QoL, depression, self‐efficacy, pain, and fatigue. These findings will help identify the most promising components of SMIs, guiding targeted interventions for specific subpopulations, and facilitating the design of better interventions.

## Key points

- The effectiveness of self‐management interventions (SMIs) for multiple sclerosis (MS) remains uncertain, underscoring the importance of this study in providing clarity about their impact.
- The insights gained from this research will contribute to the creation of more effective SMIs for MS patients. By understanding which components are impactful, future interventions can be better designed and optimized.

## 1 INTRODUCTION

Multiple sclerosis (MS) is a demyelinating disease in which the sheaths of nerve cells in the brain and spinal cord are damaged. The disease typically manifests between the ages of 20 and 40, although it can occur in childhood in less than 1% of cases and approximately 2%−10% of cases after the age of 50.^1^, ^2^, ^3^ The inflammatory and pathological conditions that lead to this disease affect women about four times more than men.^4^, ^5^ The prevalence of this disease in the world is 2.3 million people.^6^ The signs and symptoms of the disease are unpredictable and uncertain, leading to various physical and psychological outcomes. These outcomes include depression, fatigue, optic neuritis, tremor, gait disturbance, and pain, which can significantly impact the quality of life (QoL) for these patients.^7^, ^8^, ^9^ MS often occurs during the years when a person expects good health, and the onset of this disease can distort both their body image and overall health.^10^, ^11^ Many studies today have expressed the need for comprehensive self‐management programs and preventive strategies such as educational programs to improve well‐being and reduce the symptoms of chronic diseases.^12^, ^13^ Self‐management can be defined as “*the individual's ability to manage the symptoms, treatment, physical and psychosocial consequences, and lifestyle changes inherent in living with a chronic condition.”* ^14^ This program is a set of regular activities to help patients with chronic diseases that lead to active participation and control of factors involved in the disease and includes self‐control of symptoms and physiological process of the disease, decision‐making, and management of the disease and its complications.^15^, ^16^ Nurses have consistently fulfilled a crucial role in patient education. Numerous studies have highlighted the nurse as one of the foremost, highly effective, and essential educators for patients. Moreover, substantial evidence supports the nurse's pivotal role as a teacher and in overseeing educational programs to enhance self‐management among patients with chronic illnesses.^17^, ^18^, ^19^ The nurse plays an important role in self‐management programs due to their direct contact with patients. This role offers an opportunity to alleviate the burden of the disease on patients, families, and healthcare systems by preventing disease recurrence and reducing the severity of complications.^20^, ^21^ During the search across different databases, several clinical trial studies were conducted to investigate the effect of self‐management interventions (SMIs) in improving the health outcomes of people with MS (PwMS). These studies examined various outcomes such as QoL, depression, fatigue, pain, and anxiety.^22^, ^23^, ^24^, ^25^, ^26^ SMIs have been implemented in various ways, such as educational interventions delivered through face‐to‐face interactions, digital‐based education, and web‐based platforms. The findings suggest the positive impact of SMIs on enhancing certain outcomes for PwMS. However, it is noteworthy that certain studies have not yielded consistent results.^27^, ^28^, ^29^ The most recent review study in this field is from 2017, which highlighted the need for a systematic review with well‐defined inclusion criteria and an expanded search approach. Additionally, the study emphasized the importance of exploring further outcomes to enhance our understanding in this area.^30^ The purpose of this systematic review is to determine the effectiveness of SMIs on health‐related outcomes of PwMS by reviewing the relevant randomized controlled trials (RCTs) and also identifying the limitations and future research needs.

## 2 METHODS

### 2.1 Aims

The objective of this review is to conduct a systematic review and meta‐analysis to estimate the effectiveness of SMIs on outcomes of PwMS such as fatigue, depression, pain, self‐efficacy, and QoL.

### 2.2 Review questions

How effective are SMIs in reducing depression of PwMS?

How effective are SMIs in improving the QoL of PwMS?

How effective are SMIs in improving the self‐efficacy of PwMS?

How effective are SMIs in reducing pain of PwMS?

How effective are SMIs in reducing fatigue of PwMS?

### 2.3 Methodology

This systematic review will be performed according to the procedures outlined in the Cochrane Handbook^31^ and also Preferred Reporting Items for Systematic Reviews and Meta‐Analyses (PRISMA),^32^ as well as Preferred Reporting Items for Systematic Reviews and Meta‐Analysis Protocols (PRISMA‐P).^33^, ^34^ This protocol has also been registered at the Open Science Framework with the registration link as https://osf.io/92xdq.

### 2.4 Criteria for study selection

Inclusion criteria for this study are as follows:

We will include RCTs that describe the effectiveness of SMIs on outcomes of PwMS, such as fatigue, depression, pain, self‐efficacy, and QoL, without any restrictions on the age and sex of patients. Published articles in any language will be considered, as long as they have full English abstracts.

The components of the PICO based on the questions in the present study are: P (population): PwMS whose diagnosis has been confirmed; I (intervention): The intervention includes a variety of SMIs, including face‐to‐face training, mobile‐based training, telephone, digital, and so forth which includes all three key tasks of self‐management (medical, emotional, and role management)^14^, ^21^; C (comparison): The control group includes patients who have not received any interventions or not SMIs; O (outcome): The main outcomes include fatigue, depression, QoL, pain, and self‐efficacy.

Exclusion criteria for this study are as follows:

1. Duplicate publications of the same material will be excluded. If a study has been published in multiple journals or conferences, the most recent and comprehensive version will be selected for inclusion.
2. Non‐RCTs and non‐interventional articles such as observational studies, narrative reviews, opinion pieces, letters, and any other publications lacking primary data and/or explicit method descriptions will be excluded.
3. Studies on SMIs that do not include the three key tasks of self‐management (medical, emotional, and role management) will be excluded.
4. Trials that include populations other than MS patients or have mixed populations will be excluded.

### 2.5 Data sources and search strategy

Electronic searches: We will conduct a search using relevant keywords such as “MS” and “self‐management” in databases including PubMed, SCOPUS, EMBASE, ISI Web of Science, PsyINFO, and the Cochrane Central Register of Controlled Trials (CENTRAL). The search will encompass the period from 2000 to July 2023, with no restrictions on language.

### 2.6 Other resources

- Reference lists of relevant primary studies, reviews, and key journals
- Gray literature resources include: Google Scholar, Open Gray, ProQuest, Scopus, contact with experts, conferences, protocol databases like ClinicalTrials.gov, International Standard Randomized Controlled Trial Number (ISRCTN), and The WHO International Clinical Trials Registry Platform (ICTRP).

### 2.7 Medline search strategy

MeSH tags were found in Medline. The search strategies have been conducted based on the PICOS framework. The details of the Medline database search syntax are provided below (Table 1).

**Table 1:** MEDLINE search strategy.

S1: self*care.ab.ti
S2: self*manag*.ab.ti
S4: self*monitor*.ab.ti
S4: self*help.ab.ti
S5: self*regulat*.ab.ti
S6: OR/S1‐S5
S7: (MH “multiple sclerosis + “)
S8: (MH “myelitis”)
S9: (MH “myelitis, transverse”)
S10: (MH “demyelinating autoimmune diseases CNS”)
S11: (MH “multiple sclerosis, chronic progressive”)
S12: (MH “multiple sclerosis, relapsing remitting”)
S13: multiple N6 sclerosis ti.ab
S14: (MH “disseminated sclerosis”)
S15: OR/S6‐S13
S16: S6 and S15
Ti: title word
Ab: abstract word
MH: Main index/MeSH term

*Note*: The search syntax will be modified in other databases.

### 2.8 Screening procedures of eligible studies

**Figure 1:**
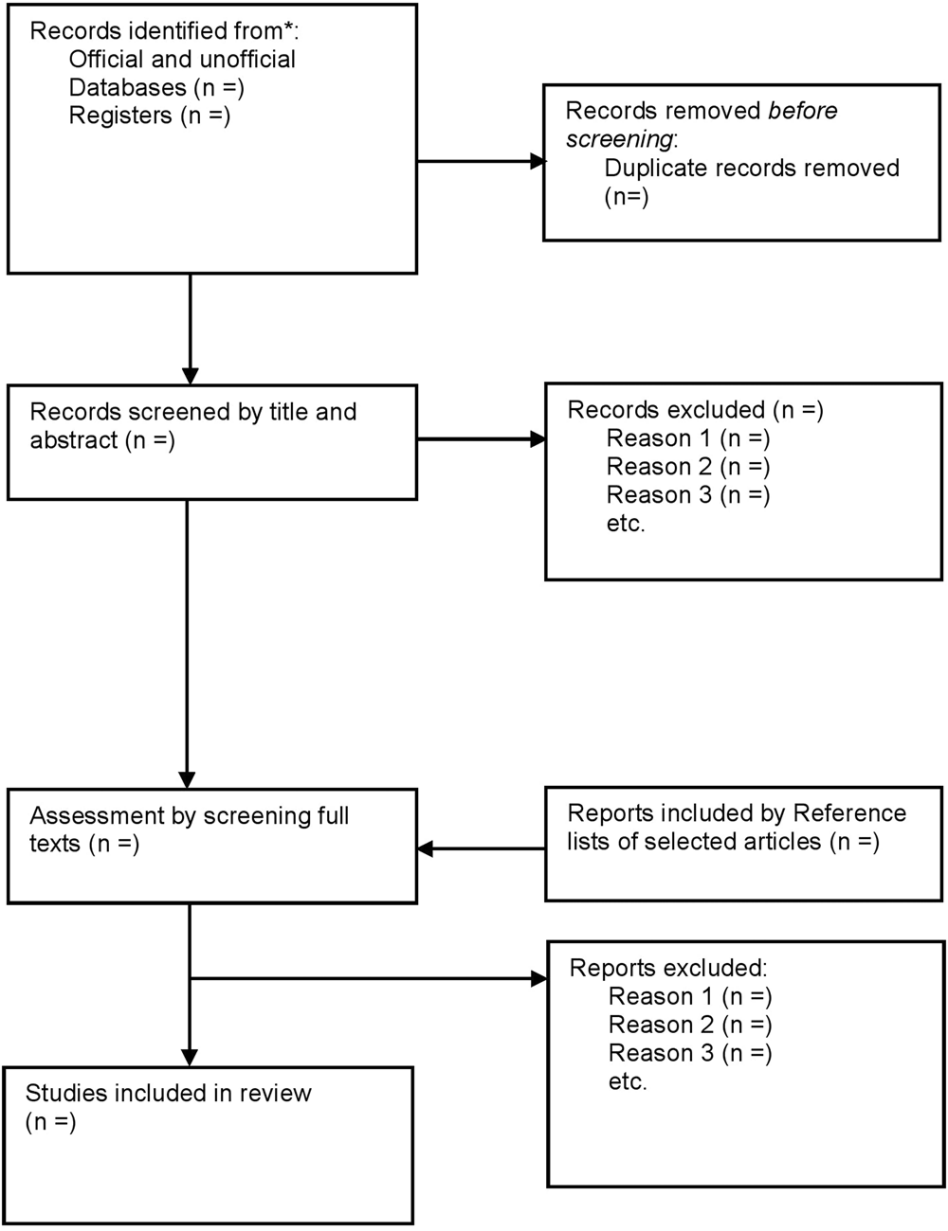
Plan of study screening and selection process.

After the initial systematic search, results will be imported into Endnote software version X8. All duplicate articles will be identified and finally removed. in the screening phase, the titles and abstracts of the articles will be screened by two reviewers (R. H. S. and A. R.) independently according to the inclusion criteria. In the screening phase; for the eligible titles and abstracts, corresponding articles will be obtained in full text and also independently assessed to select the articles which meet all the inclusion criteria. Any discrepancies will be resolved by consensus and if the disagreement is not resolved a third author (A. N. N.) will do the final assessment. The plan of study screening and selection is available in Figure 1 (adapted from an updated guideline for reporting systematic reviews).

### 2.9 Assessment of risk of bias

The methodological quality of all full‐text manuscripts included in the review will be assessed by two authors independently according to the up‐to‐date guidelines from the Cochrane Handbook for evaluating the risk of bias of included RCTs.^31^, ^35^ RCTs quality will be assessed for five domains: selection bias, performance bias, detection bias, attrition bias, reporting bias, and other bias. Any inconsistencies will be resolved by consensus if the disagreement is not resolved, the opinion of a third expert will determine the case (M. Z. or A. N. N.). Risk of bias will then be categorized as high, low, or unclear if the data are uncertain or insufficient.

### 2.10 Data extraction

Data extraction will be conducted by two authors (R. H. S. and A. R.) using a predesigned data extraction form which will include the basic outline of the evidence under study such as aims, primary/secondary outcomes, sample characteristics, intervention content, length of follow‐up, analysis methods, results necessary to support critical appraisal, intervention effectiveness, and study limitations. Discrepancies will be resolved by the mentioned method. In the absence of the required data in the included studies, the authors will attempt to collect them by using a WebPlotDigitizer or communicate with their corresponding authors to obtain the data, if the author fails to respond three times the study will be removed.

### 2.11 Missing data management

The missing or unclear data will be obtained by contacting the authors via email if possible. We will analyze the available data, and sensitivity analysis will be conducted to check the robustness of the results.

### 2.12 Strategy for data synthesis

Descriptive statistics will be calculated using SPSS 16 (IBM Company) to report the characteristics of the references, information on the authors, publication year, population, interventions, settings, and so forth. Given that the studies will vary a great deal in terms of the design, method, and outcome measures, the primary strategy for data synthesis will be narrative/descriptive. The Robvis (Risk‐of‐Bias Visualization) package will be used to draw Risk‐of‐Bias figures.^36^

If possible, meta‐analysis of this study will be performed using random‐effect models based on mean differences and their standard deviation of fatigue, depression, mental, and physical components of QoL, pain, and self‐efficacy. The effect size will be calculated by using Cohen's *d* statistic,^37^ in cases where the standard deviation is not reported directly, Hozo et al. method will be used depending on the results of the articles.^38^, ^39^ To calculate the overall effect size, a random‐effect model will be used to consider heterogeneity between studies.^40^ To determine heterogeneity, *I* ^2^ statistics (values higher than 50% will be considered as high heterogeneity) and *Q*‐test (with values less than 0.05) will be used.^41^ Funnel plot as well as Egger's and Begg's test will be used for publication bias.^42^ For sensitivity analysis of the studies, the leave‐one‐out method will be used.^43^ All statistical analyses will be conducted by using STATA software (StataCorp.) version 14.2.

### 2.13 Validity, reliability, and rigor

We will attempt to use the GRADE (Grading of Recommendations Assessment, Development and Evaluation) approach for creating a summary of findings and also all reporting and design of our systematic review will follow the requirements of the Cochrane Handbook^31^ and its protocol was completed based on PRISMA‐P^33^, ^34^ to ensure the validity and reliability.

## 3 DISCUSSION

SMIs designed to improve health‐related outcomes have been widely implemented in clinical trials for PwMS despite the efficacy of these interventions remains uncertain. This systematic review with meta‐analyses will enable us to identify whether SMIs improve outcomes in PwMS or not. It will provide evidence with reliable data by synthesizing research, identifying, and combining information, and will simplify how to implement it for professionals involved in health services, public health, and public policy. This study will enable us to analyze, identify, and select SMIs that consider the three tasks of the self‐management model. It will compare the efficacy of SMIs, provide evidence on the most effective interventions, and identify and compare them to support the development of efficient guidelines and the adoption of appropriate strategies for educational programs. Our results will identify the most effective intervention for each outcome of PwMS and can support nurses and clinicians in choosing the efficient approach.

## AUTHOR CONTRIBUTIONS

**Reza Heidari‐Soureshjani**: Conceptualization; formal analysis; investigation; methodology; project administration; software; writing—original draft. **Alireza Nikbakht Nasrabadi**: Conceptualization; methodology; validation; writing—review and editing. **Masoumeh Zakerimoghadam**: Conceptualization; investigation; validation; writing—review and editing. **Tayeb Mohammadi**: Formal analysis; investigation; methodology; software; visualization; writing—review and editing. **Arezoo Rasti**: Conceptualization; investigation; methodology; supervision; validation; writing—review and editing.

## CONFLICT OF INTEREST STATEMENT

The authors declare no conflict of interest.

## ETHICS STATEMENT

Ethical approval for this study was obtained from ethics committee of Tehran University of Medical Sciences (TUMS), (Approval ID: IR.TUMS.FNM.REC.1399.074). Participant consent and ethical approval were not required for this research because this research is being conducted using systematic review methods with existing RCTs data.

## TRANSPARENCY STATEMENT

The lead author Arezoo Rasti affirms that this manuscript is an honest, accurate, and transparent account of the study being reported; that no important aspects of the study have been omitted; and that any discrepancies from the study as planned (and, if relevant, registered) have been explained.

## Data Availability

Data sharing is not applicable to this article as no data were generated during the current study.
